# Phase I dose-escalation trial of S-1 combined with carbon-ion radiotherapy for sinonasal squamous cell carcinoma

**DOI:** 10.1093/jrr/rraa037

**Published:** 2020-07-09

**Authors:** Daiki Takahashi, Yusuke Demizu, Sung Chul Park, Yoshiro Matsuo, Nor Shazrina Sulaiman, Kazuki Terashima, Sunao Tokumaru, Masaya Akashi, Tomoaki Okimoto

**Affiliations:** 1 Department of Radiology, Hyogo Ion Beam Medical Center, 1-2-1 Koto, Shingu-cho, Tatsuno, Hyogo 679-5165, Japan; 2 Department of Radiation Oncology, Hyogo Ion Beam Medical Center Kobe Proton Center, 1-6-8 Minatojima-minamimachi, Chuo-ku, Kobe, Hyogo 650-0047, Japan; 3 Department of Oral and Maxillofacial Surgery, Kobe University Graduate School of Medicine, 7-5-2 Kusunoki-cho, Chuo-ku, Kobe, Hyogo, Japan, 650-0017

**Keywords:** Carbon-ion radiotherapy, S-1, concomitant chemoradiotherapy, head and neck cancer, sinonasal cancer, squamous cell carcinoma

## Abstract

This study aimed to determine the maximum tolerance dose (MTD) and to estimate the recommended dose (RD) of concomitant S-1 with carbon-ion radiotherapy (RT) for sinonasal squamous cell carcinoma (SCC). Nine patients with sinonasal SCC received carbon-ion RT with escalating doses of S-1 according to phase I methods. Doses of 40, 60 and 80 mg/m^2^/day were administered twice daily in dose levels 1, 2 and 3, respectively, from days 1 to 14 and 22 to 35. Carbon-ion RT was administered at a dose of 70.4 Gy (relative biological effectiveness) in 32 fractions, 5 days a week. Two patients developed grade 3 acute dermatitis. However, none developed dose-limiting toxicities. Therefore, the MTD of S-1 could not be determined; the RD was estimated to be 80 mg/m^2^/day with concurrent carbon-ion RT. Partial response and stable disease were noted in 5 and 4 patients, respectively. The 2-year overall survival and local control rates were 56 and 74%, respectively. Overall, 2 patients developed ≥grade 3 late toxicities; among them, 1 patient developed grade 3 cataract and the other developed grade 4 cataract, optic nerve disorder and hearing impairment. To the best of our knowledge, this phase I study is the first clinical trial to evaluate concomitant S-1 with carbon-ion RT for sinonasal SCC. The MTD of S-1 could not be determined, and the RD was estimated to be 80 mg/m^2^/day. This study demonstrated a manageable safety profile for this combination. The observed outcomes may facilitate further evaluation of this novel therapy.

## INTRODUCTION

Sinonasal cancers include malignancies of the nasal cavity and paranasal sinuses, and are typically rare, with an average annual incidence ranging between 5 and 10 per million and 2 and 5 per million in males and females, respectively [1]. Sinonasal cancers comprise various histologies, including squamous cell carcinoma (SCC), adenoid cystic carcinoma (ACC), adenocarcinoma (AC) and malignant melanoma (MM), among others. SCC is the most common histological type of sinonasal cancer [2]. Surgery, chemotherapy and radiotherapy (RT) are essential components of treatment in SCC of the head and neck, including sinonasal SCC [3]. Surgery with postoperative concurrent chemoradiotherapy is currently one of the most successful treatment approaches [4]. However, definitive RT is often offered to patients with advanced sinonasal cancers that are considered unresectable.

In December 1904, William Henry Bragg published data on the curves for ionization by alpha particles from radium. He demonstrated that the density of ionization increased sharply near the distal extent of the range [5]. Particle therapy-based modalities, including protons and carbon-ions demonstrate the advantage of a rapid dose fall-off at the distal end, known as the Bragg peak. They can achieve superior dose distributions compared with photons [6]. At the National Institute of Radiological Sciences (NIRS) in Japan, carbon-ion RT was initiated in 1994. Compared with photons and protons, carbon-ions provide a higher linear energy transfer and greater relative biological effectiveness (RBE). The dose-localizing properties of carbon-ions establish their superiority over photons and protons [7].

Reports on clinical outcomes in patients who underwent particle therapy, including carbon-ion RT alone for sinonasal SCC [8], have suggested that this treatment modality may play an important role in treating sinonasal SCC. The 5-year local control (LC) rate was 50.4%, but this result is lower than LC rates of patients treated with carbon-ion RT for ACC (68%), AC (79.3%) and MM (72.3%) of the head and neck [9–11]. There was a need to get better LC in this treatment. The efficacy and safety of carbon-ion RT with concomitant chemotherapy remains unknown. S-1 is a combination drug comprising tegafur, gimeracil and oteracil potassium, and is considered essential in the control of head and neck cancers [12]. Reports have suggested that gimeracil enhances the efficacy of radiation via suppression of homologous recombination DNA repair [13]. Concurrent chemoradiotherapy using photons and S-1 has been reported to be effective in patients with head and neck SCC [14]. This study aimed to determine the maximum tolerance dose (MTD) and to estimate the recommended dose (RD) of concomitant S-1 with fixed dose carbon-ion RT for sinonasal SCC.

## MATERIALS AND METHODS

The institutional review board reviewed and approved the protocol, and the study was performed in accordance with the principles of the Declaration of Helsinki. All patients provided written informed consent. This study is registered with the University Hospital Medical Information Network Clinical Trials Registry (http://www.umin.ac.jp/ctr/index-j.htm), identification number: UMIN000012135.

### Eligibility criteria

Patients with histologically proven, grossly measurable sinonasal SCC were eligible. The other inclusion criteria were an Eastern Cooperative Oncology Group performance status of 0 or 1, age 20–75 years, ability to be immobilized using a standard head immobilization system for ~30 min, sufficient oral intake, adequate organ function and no abnormal electrocardiogram within 28 days prior to registration.

Patients were excluded from this study if they had a history of RT for the primary site, pulmonary fibrosis or interstitial pneumonia detected by chest radiographs within 28 days prior to registration, watery diarrhea, active infections except viral hepatitis, serious comorbidities (including heart failure, renal failure, liver failure, hemorrhagic peptic ulcer, paresis of the intestine, intestinal obstruction and severe diabetes), moderate or severe ascites or pleural effusion, central nervous system metastases, active synchronous or metachronous double cancers within the 3 years prior to registration (carcinoma *in situ* and intramucosal carcinoma were permitted), or were undergoing treatment with flucytosine, phenytoin or warfarin potassium. In addition, female patients who were/could be/wished to get pregnant or were breast-feeding, and male patients whose partners were planning to get pregnant, were excluded. Those with severe mental disorders, whom physicians judged to be ineligible for this study, were also excluded.

### Study design, dose escalation and treatment

**Fig. 1.**
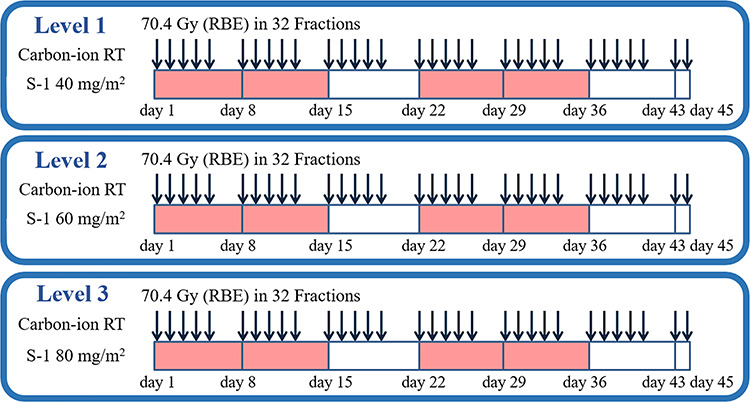
Treatment scheme of S-1 combined with carbon-ion radiotherapy for sinonasal squamous cell carcinoma

This study was designed to determine the MTD and to estimate the RD of concomitant S-1 with a fixed dose of carbon-ion RT for sinonasal SCC. S-1 was administered twice daily from days 1 to 14 and 22 to 35. Three dose-levels of 40, 60 and 80 mg/m^2^/day were investigated at dose levels 1, 2 and 3, respectively (Fig. 1).

The patients received treatment with 320 or 375 -MeV/n carbon-ion beams. The radiation treatments were planned on the XiO-M (Mitsubishi Electric, Tokyo, Japan) radiotherapy planning system. The doses of the carbon-ion beam were calculated as the photon-equivalent doses in Gray (RBE). The RBE values for this beam were determined based on previously published radiobiological data [15]. The RBE values for carbon-ion RT were between 2 and 3.7 (depending on the spread-out Bragg peak of the depth dose distributions). All patients received a total dose of 70.4 Gy (RBE) in 32 fractions for 5 days a week. Each patient was immobilized using a custom-made thermoplastic cast. Computed tomography (CT) and magnetic resonance imaging (MRI) scans were performed with slice thicknesses of 1 and 1–6 mm, respectively. The target volumes and organs at risk were delineated on the CT–MRI fusion images. The clinical target volume (CTV) was defined as the gross tumor volume (GTV) with a 5-mm basic margin; the adjacent structures were included in selected patients. The planning target volume (PTV) was defined as the CTV with an additional setup margin of 3 mm.

The evaluation for dose-limiting toxicities (DLTs) and other toxicities was performed on day 45, based on the Common Terminology Criteria for Adverse Events (CTCAE), version 4.0. DLTs were defined as follows: grade 4 hematological toxicities, oral mucositis or dermatitis; fever with temperatures >38°C with grade 3 neutropenia; grade 3 non-hematologic toxicities except oral mucositis, dermatitis, anorexia, nausea or alopecia; reduction of S-1 doses to less than two-thirds of the planned dose; or suspension of carbon-ion RT for at least 2 weeks.

Initially, 3 patients received level 1 doses. The dose was escalated to the next level when no DLTs were observed. In the case of DLT occurrence, an additional 3 patients were required. On confirmation of DLTs in 3/6 patients or fewer, the dose was escalated to the next level. The MTD was defined as the dose of S-1 that produced DLTs in more than 4/6 patients. In principle, the RD was 1 level below the MTD. If the MTD was not obtained at level 3, the dose at this level was considered the RD.

Tumor response was evaluated according to the Response Evaluation Criteria in Solid Tumors (RECIST), version1.1. Overall survival (OS) and LC were defined as the duration between the initiation of chemoradiotherapy and death and relapse, respectively, according to the Kaplan–Meier method.

## RESULTS

### Patients

Among 9 patients enrolled and dosed in this study, 5, 2, 1 and 1 had SCC of the maxillary sinus, ethmoidal sinus, sphenoidal sinus and nasal cavity, respectively. The baseline characteristics of the 9 patients are presented in Table 1. Because patient level 2 No. 2 was a young female, she decided not to undergo surgery for cosmetic reasons. The tumor was close to her eyeball and optic nerve, therefore she received neoadjuvant chemotherapy (NAC) to improve the coverage of carbon-ion therapy. The tumor in patient level 3 No. 3 could not be resected with a safety margin. She also decided not to undergo surgery for cosmetic reasons.

**Table 1:** Patient characteristics

Level	No.	Age	Gender^a^	Primary site	TNM	Stage	NAC	Tumor response to NAC	GTV (mL)
1	1	57	M	Maxillary sinus	T4bN0M0	IVB	CDDP +5-FU + IA-CDDP	SD	38
	2	69	M	Maxillary sinus	T4aN0M0	IVA	CDDP +5-FU	PR	48
	3	68	M	Maxillary sinus	T4bN0M0	IVB	S-1 + NDP	SD	161
2	1	62	M	Ethmoid sinuses	T4aN0M0	IVA	DTX + CDDP +5-FU	PD	68
	2	27	F	Nasal cavity	T2N0M0	II	NDP + 5-FU	SD	21
	3	70	M	Sphenoid sinus	N/A^b^	N/A^b^	DTX + CDDP +5-FU	SD	132
3	1	66	M	Maxillary sinus	T4bN0M0	IVB	−	−	187
	2	61	M	Ethmoid sinuses	T4bN0M0	IVB	−	−	79
	3	49	F	Maxillary sinus	T2N0M0	II	−	−	13

NAC, neoadjuvant chemotherapy; GTV, gross tumor volume; CDDP, cisplatin; 5-FU, fluorouracil; IA-CDDP, intra-arterial infusion of CDDP; NDP, nedaplatin; DTX, docetaxel; PR, partial response; SD, stable disease; PD, progressive disease; N/A, not available. ^a^M, male; F, female; ^b^with invasion into the skull base, classifying it as T4bN0M0, which is equivalent to stage IV.

The median age of the cohort was 62 years (range: 27–70 years) and the median GTV was 68 mL (range: 13–187 mL). Overall, 6 and 2 patients were diagnosed with stages IV and II disease, respectively; the tumor in the other patient with SCC of the sphenoidal sinus invaded into the skull base, classifying it as T4bN0M0, which is equivalent to stage IV. Six patients had received NAC. The NAC regimens included cisplatin (CDDP) + fluorouracil (5-FU) + intra-arterial infusion of CDDP (IA-CDDP), CDDP +5-FU, S-1 + nedaplatin (NDP), docetaxel (DTX) + CDDP +5-FU, and 5-FU + NDP. Partial response (PR), stable disease (SD) and progressive disease (PD) were observed in 1, 4 and 1 patients, respectively.

### Toxicities

The observed toxicities are listed in Table 2. Two patients developed grade 3 acute dermatitis. However, none of the patients in this cohort developed DLTs. Therefore, the MTD of S-1 was not determined; the RD was estimated to be 80 mg/m^2^/day with concurrent carbon-ion RT. Two patients developed ≥grade 3 late toxicities, of whom 1 developed grade 3 cataract and the other developed grade 4 cataract, optic nerve disorder and hearing impairment.

**Table 2:** Acute and late toxicities

Level	No.	Primary site	TNM	Stage	DLT	Acute dermatitis	Acute mucotitis	Late toxicity (grade ≥ 3)
1	1	Maxillary sinus	T4bN0M0	IVB	−	2	2	−
	2	Maxillary sinus	T4aN0M0	IVA	−	2	2	−
	3	Maxillary sinus	T4bN0M0	IVB	−	2	2	−
2	1	Ethmoid sinuses	T4aN0M0	IVA	−	2	1	−
	2	Nasal cavity	T2N0M0	II	−	3	1	Cataract (grade 3)
	3	Sphenoid sinus	N/A	N/A	−	2	2	−
3	1	Maxillary sinus	T4bN0M0	IVB	−	3	1	Cataract (grade 4), Optic nerve disorder (grade 4), hearing impairment (grade 4)
	2	Ethmoid sinuses	T4bN0M0	IVB	−	1	0	−
	3	Maxillary sinus	T2N0M0	II	−	2	1	−

DLT, dose-limiting toxicity.

**Fig. 2.**
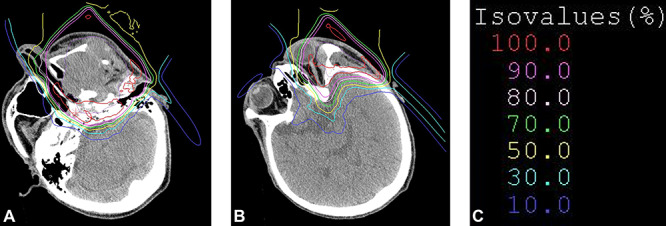
Treatment plan in patient level 3 No. 1. (**A**, **B**) Dose distribution in axial view. (**C**) Isovalues.

The treatment plan in patient level 3 No.1, with axial images, is shown in Fig. 2. In this case, the probability of blindness and hearing loss was predictable, and carbon-ion RT was performed with sufficient informed consent prior to the treatment. Grade 4 cataract, optic nerve disorder and hearing impairment occurred only on the affected side. Because the patient had eyesight and hearing on the unaffected side, he could continue working without significant reduction of quality of life 3 years after the treatment.

### Treatment efficacy

**Fig. 3.**
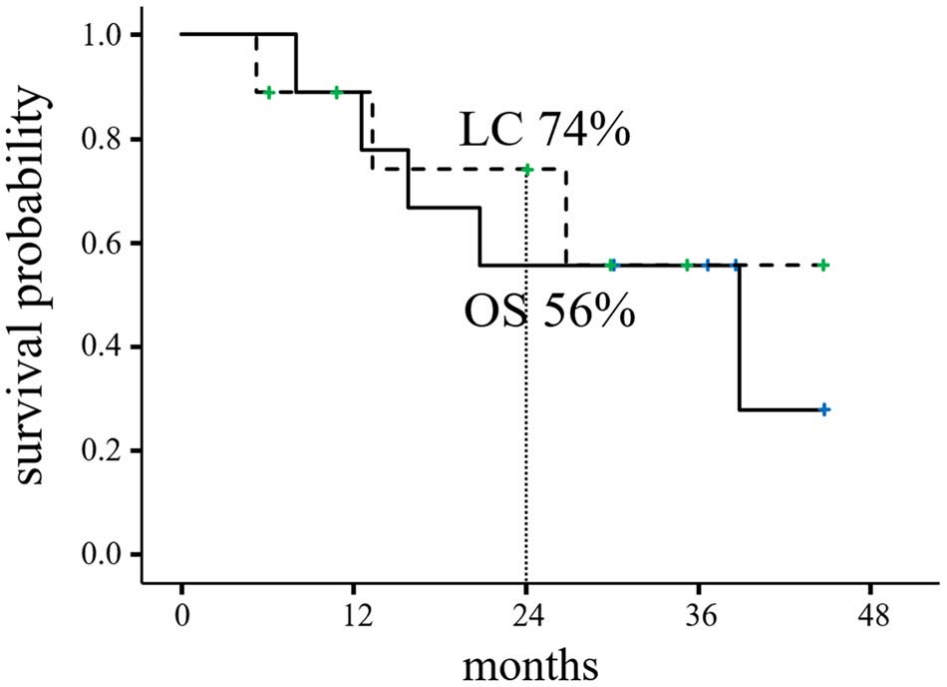
Kaplan–Meier estimates of overall survival (OS) and local control (LC).

The clinical outcomes are presented in Table 3 and Fig. 3. Among the 9 patients in this study, PR and SD were noted in 5 and 4 patients, respectively. Two patients had received adjuvant chemotherapy (AC); the AC regimens included DTX + CDDP +5-FU and S-1. The median duration of follow-up was 30.1 months (range: 8.0–44.8 months). The 2-year rates for OS and LC were 56 and 74%, respectively. Overall, 3 and 1 patients received chemotherapy and photon RT for recurrences, respectively. The chemotherapy regimens for recurrences included carboplatin (CBCDA) + paclitaxel (PTX) + cetuximab (Cmab) and PTX + Cmab.

**Table 3:** Clinical outcomes

Level	No.	TNM	Tumor response	AC	Vital status	Follow-up period (months)	Local reccurence	Local control (months)	Distant metastasis	Treatment after recurrence
1	1	T4bN0M0	SD	−	Dead	15.8	+	5.2	−	CBDCA + PTX + Cmab
	2	T4aN0M0	SD	−	Dead	12.6	−	10.8	Contralateral maxillary sinus, cervical lymph node, lung, liver	CBDCA + PTX + Cmab
	3	T4bN0M0	PR	−	Dead	8.0	−	6.1	Lung, bone	Photon RT
2	1	T4aN0M0	PR	−	Dead	20.8	+	13.3	−	No treatment
	2	T2N0M0	SD	−	Alive	44.8	−	44.7	−	−
	3	N/A	SD	DTX + CDDP +5-FU	Dead	38.9	+	26.8	−	PTX + Cmab
3	1	T4bN0M0	PR	S-1	Alive	38.6	−	35.3	−	−
	2	T4bN0M0	PR	−	Alive	36.7	−	24.1	−	−
	3	T2N0M0	PR	−	Alive	30.1	−	29.9	−	−

AC, adjuvant chemotherapy; SD, stable disease; PR, partial response; DTX, docetaxel; CDDP, cisplatin; 5-FU, fluorouracil; CBCDA, carboplatin; PTX, paclitaxel; RT, radiotherapy; Cmab, cetuximab.

## DISCUSSION

To the best of our knowledge, this phase I study is the first clinical trial to evaluate the safe dosing of concurrent S-1 with carbon-ion RT for sinonasal SCC. In this cohort, no DLTs occurred in the set dose ranges. The RD of S-1 with carbon-ion RT was considered to be 80 mg/m^2^/day.

In terms of acute toxicities, only 2 (22%) patients developed grade 3 dermatitis, and all patients completed the planned carbon-ion RT schedule. Lansu *et al*. [16] reported that in a cohort of patients receiving photon RT for sinonasal tumors, the incidence of acute mucositis, dermatitis and dysphagia ≥grade 3 was 29%. The lower incidence of acute toxicity in this study may be attributed to the superior dose distribution of carbon-ion RT and the safety of S-1. In a cohort with sinonasal SCCs, the incidence of ≥grade 3 acute dermatitis was reported to be 14% for carbon-ion RT alone [8]. In terms of acute toxicities, our treatment schedule apparently produced similar results to carbon-ion RT alone. In terms of late toxicities, 2 (22%) long-term survivors developed ≥grade 3 toxicities. Severe cataracts [17], optic nerve disorder [18], and hearing impairment [19] has often been reported with photon RT. Since the tumors in the 2 patients in our cohort were near the optic nerve, eyeball and auditory nerve, these toxicities were considered inevitable to achieve tumor control. In sinonasal SCCs, the incidence of ≥grade 3 late toxicities have been reported to be 29% for carbon-ion RT alone [8]. Therefore, our treatment schedule was associated with similar late toxicities to carbon-ion RT alone. S-1 combined with carbon-ion RT proved to be both safe and tolerable in patients with sinonasal SCC.

Although concurrent chemoradiotherapy has become the standard of care for patients with SCC of the head and neck [20], only a few reports are available on the use of concomitant chemotherapy with carbon-ion RT [21, 22]. Concomitant S-1 with carbon-ion RT has never been studied for sinonasal SCC. The S-1 dose (80 mg/m^2^/day) was based on reports of phase I studies on combination therapy with photon RT [23, 24]. Although the evaluation of efficacy was not the primary objective in this small phase I study, all 3 patients at level 3 (80 mg/m^2^/day) showed higher responses than patients at levels 1 or 2. This suggests that a higher dose of S-1 is more favorable for combination therapy with carbon-ion RT.

Late toxicity is one of the most serious issues after radiotherapy for head and neck tumors. Many studies have reported it to have profound effects on quality of life [25–27]. Our treatment schedule apparently provided survival advantages; it is likely that long-term survivors would have a higher incidence of late toxicities. As mentioned previously, the 2 long-term survivors in this cohort developed severe late toxicities. All patients in this study were delivered radiotherapy using the passive scattering method. The adoption of the active scanning method is expected to reduce the risk of late toxicities [28].

The importance of NAC in head and neck SCC has been demonstrated in recent years [29]. All 6 patients had received NAC at levels 1 and 2. However, the outcomes were poorer than expected. Consequently, all 3 patients at level 3 who did not receive NAC demonstrated long-term survival. Six patients received NAC with different regimens, therefore this study did not ensure the safety of collaboration between NAC and concurrent S-1 with carbon-ion RT.

This study had some limitations that may impact the generalizability of the results. This was a non-randomized, phase 1 study with a small sample size. Further phase II and phase III studies are warranted to evaluate clinical outcomes. Additionally, long-term observation is needed to evaluate late toxicities. Patients in this study should be carefully followed-up for appropriate evaluation of late toxicities.

In conclusion, this study, which is probably the first on concomitant S-1 with carbon-ion RT for sinonasal SCC, has demonstrated a manageable safety profile. The MTD of S-1 could not be determined and the RD was estimated to be 80 mg/m^2^/day. The observed clinical outcomes may facilitate further evaluation of this novel therapy.

## CONFLICT OF INTEREST

None declared.

## FUNDING

This work was supported by the Takeda Science Foundation (to D.T.)

## ABBREVIATIONS

SCC, squamous cell carcinoma; ACC, adenoid cystic carcinoma; AC, adenocarcinoma; MM, malignant melanoma; RT, radiotherapy; NIRS, National Institute of Radiological Sciences; RBE, relative biological effectiveness; MTD, maximum tolerance dose; RD, recommended dose; CT, computed tomography; MRI, magnetic resonance imaging; CTV, clinical target volume; GTV, gross tumor volume; PTV, planning target volume; DLTs, dose-limiting toxicities; CTCAE, Common Terminology Criteria for Adverse Events; RECIST, Response Evaluation Criteria in Solid Tumors; OS, overall survival; LC, local control; NAC, neoadjuvant chemotherapy; CDDP, cisplatin; 5-FU, fluorouracil; NDP, nedaplatin; DTX, docetaxel; PR, partial response; SD, stable disease; PD, progressive disease; AC, adjuvant chemotherapy; CBCDA, carboplatin; PTX, paclitaxel; Cmab, cetuximab.
